# Acylated flavonol glycosides from *Tagetes minuta* with antibacterial activity

**DOI:** 10.3389/fphar.2015.00195

**Published:** 2015-09-11

**Authors:** Irum Shahzadi, Mohammad M. Shah

**Affiliations:** Biotechnology Program, Department of Environmental Sciences, COMSATS Institute of Information TechnologyAbbottabad, Pakistan

**Keywords:** Asteraceae, *Tagetes minuta*, 2D NMR, acylated flavonols, antibacterial

## Abstract

Wild marigold (*Tagetes minuta*), a flowering plant of the family Asteraceae contains compounds of pharmaceutical and nutritional importance especially essential oils and flavonols. Identification, characterization of flavonols and determination of their antibacterial activity were major objectives of the current study. The isolation and purification of flavonols was accomplished using chromatographic techniques while structural elucidation was completed by LC–MS and NMR spectroscopy. The extracts and purified compounds were tested against various bacterial strains for antibacterial activity. A total of 19 flavonols were isolated from this species. Of these, 17 were of butanol and two of ethyl acetate extracts. Based on the concentration and purity, eight potential flavonols were selected and structurally elucidated. Four flavonols, 6-hydroxyquercetin 7-*O*-β-(6′′-galloylglucopyranoside; **2**), 6-hydroxykaempferol 7-*O*-β-glucopyranoside (**5**), 6-hydroxykaempferol 7-*O*-β-(6′′-galloylglucopyranoside; **7**), 6-hydroxyquercetin 7-*O*-β-(6′′-caffeoylglucopyranoside; **9**), were identified for the first time from *T. minuta*. Butanol and ethyl acetate extracts of flowers and seeds showed significant antibacterial activity against *Micrococcus leteus, Staphylococcus aureus, Bacillus subtilis*, and *Pseudomonas pikettii*. Among the isolated flavonols only **1**, **2,** and **18** were found to possess significant antibacterial activity against *M. luteus*. The extracts and purified flavonols from *T. minuta* can be potential candidates for antibacterial drug discovery and support to ethnopharmacological use.

## Introduction

The genus *Tagetes* includes 56 species (Soule, 1993), widely grown all over the world for multipurpose uses including ornamental, medicinal, cultural, nutritional and therapeutics (Vasudevan et al., 1997; Shahzadi et al., 2010). *Tagetes minuta* (wild marigold) is a potential medicinal plant of the family Asteraceae and is widely grown from temperate to tropical regions of the world in a wider range of climatic conditions (altitude range from 3000 to 11000 feet).

*Tagetes minuta* is reported to contain a number of chemical and biochemical compounds of high pharmaceutical and nutritional value (Shahzadi et al., 2010). The prominent classes of compounds present in this species are essential oils and flavonols. Majority of the studies focused on the isolation and characterization of essential oils of *T. minuta* (Singh et al., 1992; Tankeu et al., 2013). Efforts toward the identification, isolation, and characterization of flavonols in *T. minuta* have been marginal.

Flavonols are secondary metabolites which represent a class of flavonoids and are widely distributed in almost all plant groups like angiosperms, gymnosperms, mosses, liverworts, and ferns. More than 393 flavonols (Vetschera and Wollenweber, 2006) and over 1331 flavonol *O*-glycosides (Williams, 2006) were reported with complete structural elucidation. Flavonols and flavones accumulate in the epidermal cells consequent to wounding, pathogenic attack, nutrient deficiency, temperature changes, ozone irradiation, UV protection and copigmentation in flowers and fruits (Flint et al., 1985; Koes et al., 1994; Shirley, 1996; Smith and Markham, 1998; Simmonds, 2003). These also enhanced wine color through copigmentation with anthocyanins (Baranac et al., 1997; Boulton, 2001). Flavonols are natural products and preparations containing these compounds as the principal physiologically active constituents have been employed in curing many diseases in human since ages (Cushnie and Lamb, 2005). Several plant-derived flavonols have been reported to possess antibacterial activity against some super bugs, which are becoming common causes of infections in the acute and long-term care units in hospitals (Xu and Lee, 2001; Chan et al., 2014).

Previous studies on *T. minuta* focused on the chemodiversity of essential oils (Singh et al., 1992; Chalchat et al., 1995; Bansal et al., 1999; Gil et al., 2000; Kaul et al., 2005; Kiran and Kaul, 2007; Upadhyaya et al., 2010; Tankeu et al., 2013), thiophenes (Gil et al., 2002; Wang et al., 2002; Saha et al., 2012; Xu et al., 2012), and flavonoids (Abdala and Seeligmann, 1995; Tereschuk et al., 1997). Major flavonols such as patuletin, quercetin, quercetagetin, and isorhamnetin along with some of their glycosides have previously been detected in *T. minuta* (Abdala and Seeligmann, 1983; Abdala and Seeligmann, 1995). Present study aimed at isolating, characterizing and determining antibacterial activities of flavonols present in *T. minuta*.

## Materials and Methods

### Plant Material

*Tagetes minuta* L plant was collected during August–October, 2010 for chemical analysis from district Abbottabad, Pakistan. The collected plants were first identified by a taxonomist and a voucher specimen was deposited (IS/ATD/H-03) at the herbarium of the Botany Department of Post-Graduate College, Abbottabad, Pakistan. The plant material was separated and divided into three different portions; (A) flowers and seeds (5.2 kg), (B) roots (3.5 kg), and (C) leaves and stems (10 kg). These portions were air dried in shade and were crushed to fine powder by locally built grinder.

### Extraction and Fractionation

Powder of all plant parts were extracted at room temperature by maceration with methanol (5L X 3) for 72 h. The methanol extracts were concentrated under reduced pressure at 30°C to give dry marc of 356 g (A), 106.4 g (B), and 461 g (C). These crude extracts were separately suspended in distilled H_2_O and subsequently extracted with *n*-hexane, chloroform, ethyl acetate and *n*-butanol. Each fraction was dried over anhydrous sodium sulfate and evaporated to yield dried fraction HA (*n*-hexane, 105 g), fraction CA (chloroform, 39.3 g), fraction EA (ethyl acetate, 21.4 g), fraction BA (butanol, 53.6 g), and WA (aqueous, 120.7 g) from flower and seeds extract and fractions, HB (*n*-hexane, 10.7 g), CB (chloroform, 3.8 g), EB (ethyl acetate, 1.3 g), BB (butanol, 1 g), and WB (aqueous, 67.6 g) from roots extract and fractions HC (chloroform, 146.4 g), CC (chloroform, 118.6 g), EC (ethyl acetate, 16.9 g), BC (butanol, 17.9 g), and WC (aqueous, 131.6 g) from leaves and stem extract. The crude extracts were defatted by *n*-hexane therefore fractions HA, HB, HC and in addition aqueous fractions were also not used for further analysis.

Fractions EA (ethyl acetate, 21.4 g) and BA (butanol, 53.6 g) were separately applied to a Sephadex LH-20 column (Pharmacia, 5 cm × 50 cm) and eluted with methanol. Eighteen fractions (∼200 mL each) were collected and evaporated to dryness under reduced pressure. Fractions 1–10 containing flavonol constituents were combined, owing to their TLC similarities, and subsequently purified by semi preparative HPLC.

### Isolation of Flavonols – Preparative HPLC

Various chloroform, ethyl acetate and butanol fractions were analyzed by high performance liquid chromatography (HPLC). Samples were filtered through a 0.45 μm Millipore membrane filterprior to injection.

The butanol and ethyl acetate fractions of flowers and seeds were subjected to preparative HPLC by (A) H_2_O-TFA (0.5%) and (B) Acetonitril-TFA (0.5%) solvents for the isolation of flavonoids using a Gilson 305/306 pump equipped with C_18_ reversed phase column [ODS-Hypersil column (25 cm × 2.2 cm, 5 μm)] coupled to a multidiode array detector (HP-1040 A; Supelco, Bellefonte, PA, USA). The elution profile consisted of a linear gradient from 10% B to 100% B for 40 min, isocratic elution (100% B) for the next 3 min, followed by the linear gradient from 100% B to 10% B for 5 min. The flow rate was 15 mL/min, and aliquots of 500 μL were injected.

### Reversed Phase Analytical HPLC-DAD

The Agilent 1100 HPLC system was equipped with a HP 1050 diode-array detector and a 200 mm × 4.6 mm, 5 μm ODS Hypersil column (Supelco, Bellefonte, USA). The elution system was binary, with A, water (0.5% TFA), and B, acetonitrile (0.5% TFA) solvents. The elution profile for HPLC consisted of initial conditions with 90% A and 10% B followed by linear gradient elution for the next 18 min to 20% B and the subsequent linear gradient conditions: 18–26 min (to 23% B), 26–30 min (to 28% B), 30–40 min (to 100% B), isocratic elution 40–43 min (100% B), and final linear gradient elution 43–48 min (to 10% B). The flow rate was 1.0 mL/min, and aliquots of 20 μL were injected with an Agilent 1100 series micro auto sampler. The UV absorption spectra were recorded on-line during HPLC analysis over the wavelength range of 280–360 nm in steps of 2 nm.

### Spectroscopy

The NMR experiments of heteronuclear multiple bond correlation (^1^H-^13^C HMBC), 2-D heteronuclear single quantum coherence (^1^H-^13^C HSQC) and 2-D homonuclear correlation experiment (^1^H-^1^H DQF-COSY), were carried out at 600.13 MHz and 150.90 MHz for ^1^H and ^13^C, respectively, on a Bruker Biospin Ultrashield Plus AV-600 MHz instrument (Fallanden, Switzerland) at 298 K. The deuteriomethyl ^13^C signal and the residual ^1^H signal of the solvent CD_3_OD were used as secondary references (δ 49.0 and δ 3.40 from TMS, respectively).

High-resolution LC-electrospray mass spectrometry (ESI^+^/TOF), spectra of **1**–**19** were recorded using a JEOL AccuTOF JMS-T100LC instrument in combination with an Agilent Technologies 1200 Series HPLC system. A Zorbax SB-C_18_ [50 mm × 2.1 mm (length × i.d.), 1.8 μm] column was used for separation, and combinations of two solvents A, H_2_O containing 0.5% TFA (v/v) and B, acetonitrile containing 0.5% TFA (v/v) were used for elution. The following solvent composition was used: 0–1.25 min 10–22% B (linear gradient), 1.25–5 min 22–30% B (linear gradient), 5–7 min 30% B (isocratic), 7–8 min 30–40% B (linear gradient), 8–14 min 40% B (isocratic), and 14–15 min 40–10% B (linear gradient). The flow rate was 0.4 mL/min.

### Antibacterial Assay

Gram positive strains of American Type of Culture Collection (ATCC) namely *Micrococcus luteus* (ATCC 10240), *Staphylococcus aureus* (ATCC 6538), *Bacillus subtilis* (ATCC 6633) and Gram-negative strains namely *Pseudomonas picketti* (ATCC 49129), *Salmonella setubal* (ATCC 19196) were used to assess the antibacterial activities of the isolated compounds and extracts.

### Turbidity Standard, Growth Media, and Culture Conditions

To compare the turbidity of bacterial culture McFarland (0.5 BaSO_4_) solution was used. The standard was prepared by adding 0.5 ml of 1% solution (w/v) of anhydrous BaCl_2_ to 99.5 ml of 1% solution (v/v) of H_2_SO_4_. Barium sulfate turbidity (4–6 ml) was taken in screw capped test tube and was used to compare the turbidity of bacteria. Nutrient broth medium for bacterial growth consisted of peptone (5 g/L), sodium chloride (5 g/L), starch (1 g/L), and agar (10 g/L; Oxoid, England). The nutrient agar plating medium consisted of plury peptone (5 g/L), beef extract (3 g/L), sodium chloride (8 g/L), and agar (15 g/L; Britanialab). Both media were prepared according to manufacturer’s instructions in distilled water and sterilized in autoclave at 121°C for 15 min. The bacterial cultures of selected strains were inoculated individually and incubated in a shaker at 150 rpm for overnight at 37°C.

### Antibacterial Activity

The agar well-diffusion method (Bibi et al., 2011) analyzed the samples for antibacterial activity. The autoclaved nutrient agar media was allowed to cool at 45°C and 25 mL was poured in each petri plate near flame. These petri plates were incubated overnight at 37°C in an incubator to avoid contamination. The wells were made by 6 mm sterile metallic borer at 24 mm distance from each other. Extracts and isolates were analyzed at 3 mg/mL in dimethyl sulfoxide (DMSO; Sigma-Aldrich, USA). Ampicillin (Sigma-Aldrich, USA, A2804) was used as standard antibiotic (positive control), up to 50 μg/mL and pure DMSO was used as negative control. Each well was filled with 20 μL of plant extract (60 μg), positive (Ampicillin, 20 μg) and negative (DMSO) control and labeled at the back of petri dishes. The incubation of sample plates was at 37°C in incubator for 24 h and all cultures were prepared in triplicate. After 24 h of incubation period, diameter of clear zones around each well was measured, showing no bacterial growth. The mean zone of inhibition was calculated with standard deviation procedure.

### Statistical Analysis

The experiments were conducted in duplicate and all the analyses were done in triplicate. One way ANOVA followed by the Duncan’s multiple-range test was used to determine significant (*p* < 0.05) differences among different extracts of plant material. The computer software SPSS 16.0 (SPSS, Inc., Chicago, IL, USA, package) carried out statistical analyses. The data was expressed as mean ± standard deviation (SD).

## Results and Discussion

The HPLC profiles revealed that the butanol fractions contained relatively high quantities of flavonoids as compared to the ethyl acetate fractions, while non-flavonoids compounds were identified in chloroform fractions. The roots, leaves, and stem extract profiles were not of high quality and therefore were not used for further spectroscopic analysis. The HPLC profiles of the butanol fraction (flowers and seeds) showed 17, while the ethyl acetate fraction of flowers and seeds showed two major flavonols (**Table 1**). The absorption maxima in the UV spectra of these compounds were recorded online during HPLC analysis in the 320–360 nm region indicating flavonol 7-*O*-glycosides based on quercetagetin, 6-hydroxy kaempferol, quercetin, and patuletin aglycones (Harborne et al., 1991; Llorach et al., 2003; Veitch et al., 2011).

**Table 1:** Relative amounts and on-line HPLC and high-resolution electrospray ionization mass spectral data recorded for **1–19.**

Compound	Relative amounts (%)	UV_max_ (nm)	t_R_ (min)	[M+H]^+^ m/z observed	[M+H]^+^ m/z calculated	Molecular formula
1	68.07	359	14.90	481.0963	481.0982	C_21_H_20_O_13_
2	30.18	365	16.27	633.1066	633.1092	C_28_H_24_O_17_
4	6.65	369	19.18	465.1015	465.1033	C_21_H_20_O_12_
5	24.06	345	19.98	465.1009	465.1033	C_21_H_20_O_12_
6	33.80	369	20.49	495.1137	495.1139	C_22_H_22_O_13_
7	19.34	349	21.38	617.1137	617.1143	C_28_H_24_O_16_
8	40.07	343	22.65	495.1140	495.1139	C_22_H_22_O_13_
9	29.36	359	23.10	643.1272	643.1299	C_30_H_26_O_16_
15	14.50	339	27.69	627.1348	627.1350	C_30_H_26_O_15_
16	37.42	337	28.32	657.1450	657.1456	C_31_H_28_O_16_
17	10.19	320	29.40	673. 1387	673.1405	C_31_H_28_O_17_
18	89.54	359	25.78	319.0433	319.0454	C_15_H_10_O_8_
19	72.36	371	35.42	333.0611	333.0610	C_16_H_12_O_8_

*Compounds **3, 10–14** were in very low concentration*.

**FIGURE 1:**
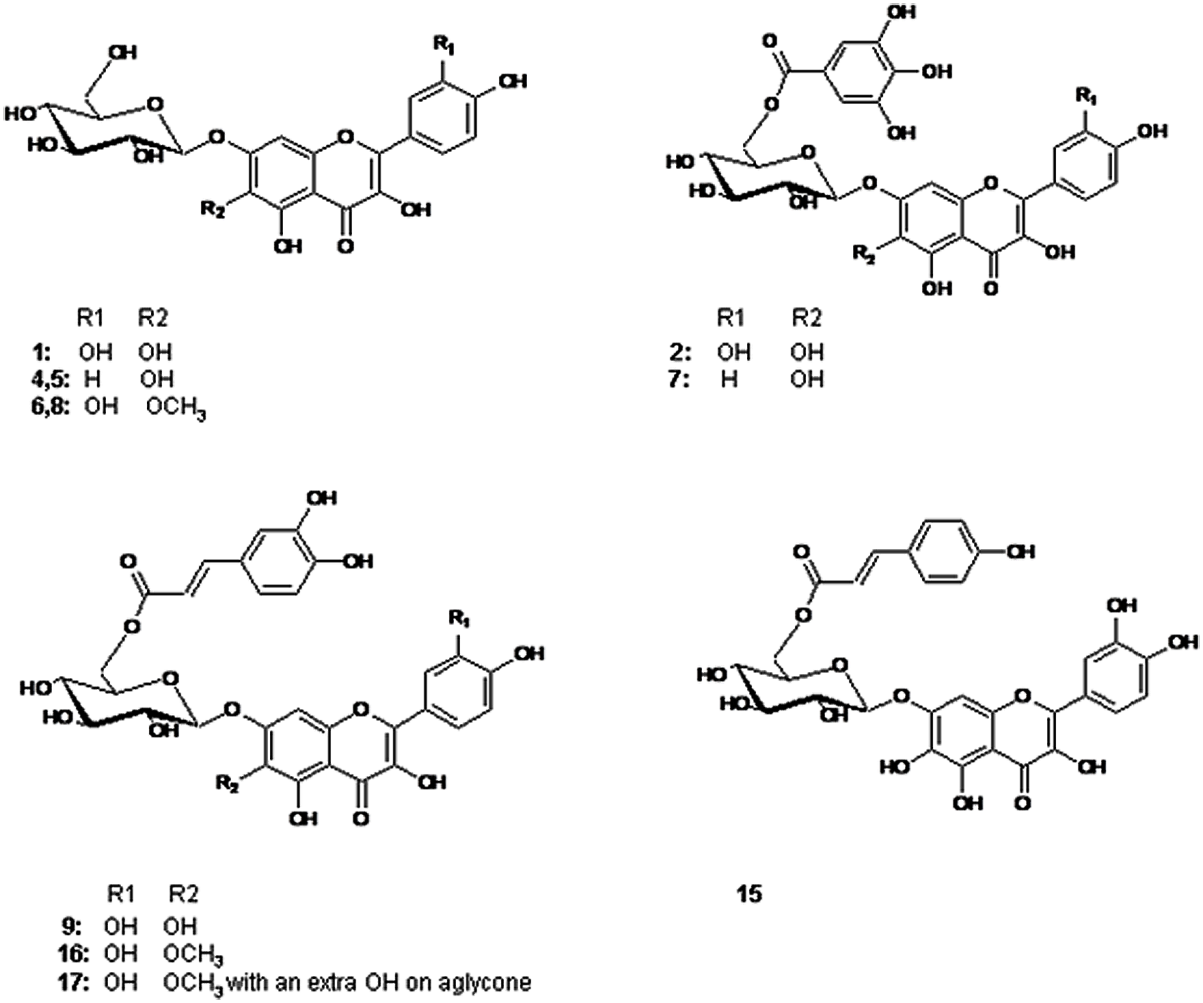
**Structures of the flavonols identified in the examined *Tagetes minuta*. 1**: 6OH Qc 7-glc, **4, 5**: 6OH Kf 7-glc, **6, 8**: 6MeO Qc 7-glc, **2**: 6OH Qc 7Gao Glc, **7**: 6OH Kf 7Gao Glc, **9**: 6OH Qc 7Caf Glc, **15**: 6OH Qc 7Cum Glc, **16**: 6MeO Qc 7Caf Glc, **17**: 6MeO Qc 7Caf Glc with a OH on aglycone. Qc = quercetin; Kf = kaempferol; Glc = glucoside; Gao = galloyl; Caf = caffeoyl; Cum = coumaroyl.

The spectroscopic analysis of **1**–**19** by UV, high-resolution LC–MS (**Table 1**), and 1D and 2D NMR (**Tables 2** and **3**) established that **2**, **4**, **5**, **7**, **9,** and **15**–**17** (**Figure 1**) were previously not been reported to occur in *T. minuta*. This is the first complete identification of compound **7** in *T. minuta*, which was tentatively identified previously (Parejo et al., 2004). Flavonols **1**, **6**, **8**, **18,** and **19** have previously been reported to occur in *T. minuta* (Abdala and Seeligmann, 1995), however, this is the first NMR characterization of **1**, **6**, **18,** and **19** isolates from *T. minuta*. Compounds **3, 10–14** were found in very low concentration for spectroscopic analysis.

**Table 2:** ^1^H NMR spectral data for **1, 2, 5–7, 9, 18, 19** in CD_3_OD at 25°C.

	1	2	5	6	7	9	18	19
***Aglycone***								
6				3.98 *s* (OCH_3_)^∗^				3.97 *s* (OCH_3_)
8	7.03 *s*	6.97 *s*^B^	7.03 *s*	6.91 *s*	6.96 *s*	6.92 *s*	6.58 *s*	6.58 *s*
2′	7.86 *d* 2.0	7.85 *d* 2.0	8.21 *d* 8.8	7.82 *d* 2.0	7.9 *d* 8.8	7.82 *d* 5.2	7.82 *d* 3.6	7.82 *d* 1.9
3′			6.99 *d* 8.9		6.92 *d* 8.9			
5′	6.98 d 8.4	6.97 *d* 8.4^B^	6.99 *d* 8.9	6.90 *d* 8.6	6.92 *d* 8.9	6.92 *d* 5.0	6.97 *d* 7.8	6.97 *d* 8.2
6′	7.76 *dd* 2.0, 8.0	7.75 *dd* 2.0, 8.5	8.21 *d* 8.8	7.52 *dd* 2.0, 8.5	7.9 *d* 8.8	7.70 *dd* 3.3, 7.5	7.72 *dd* 3.6, 7.2	7.72 *dd* 1.9, 8.0
***7-O-***β***-glucopyranosyl***								
1′′	5.14 *d* 7.4	5.20 *d* 7.4	5.14 *d* 7.5	5.19 *d* 7.6		5.18 *d* 7.4		
2′′	3.71 *m*^C^	3.71 *dd* 7.7, 9.4	3.68 *m*^B,C^	3.65 *m*^B,C^		3.72 *m*^C^		
3′′	3.65 *m*^C^	3.67 *m*^B^	3.61 *m*^B,C^	3.60 *m*^B,C^		3.66 *m*^C^		
4′′	3.53 *m*	3.68 *m*^B,C^	3.509 *t* 9.3^B^	3.510 *t* 9.3^B^		3.55 *t* 9.3		
5′′	3.66 *m*^B,C^	3.94 *m*	3.66 *m*^B,C^	3.66 *m*^B,C^		3.96 *m*^C^		
6(A)′′	4.03 *dd* 2.0, 12.0^B^	4.78 *dd* 1.9, 12.0	4.04 *dd* 2.0, 12.0^B^	4.04 *dd* 2.0, 12.0^B^		4.74 *dd* 1.9, 11.9		
6(B)′′	3.9 *dd* 6.2, 12.2^B^	4.55 *dd* 5.0, 12.0	3.81 *dd* 6.2, 12.2^B^	3.81 *dd* 6.2, 12.2^B^		4.42 *dd* 12.0, 7.2		
*6′′-O-acyl*		*6′′-O- galloyl*			*6′′-O- galloyl*	*6′′-O-caffeoyl*		
1′′′								
2′′′		7.13 *s* ^B^			7.15 *s*	6.83 *d* 1.5		
3′′′								
4′′′								
5′′′						6.62 *t* 8.1		
6′′′		7.13 *s* ^B^			7.15 *s*	6.66 *dd* 7.8, 1.8		
*α*						7.53 *d* 15.8		
β						6.27 *d* 15.8		

*^∗^Methoxy. ^B^Overlapped by other signal. ^C^From COSY. s = singlet, d = doublet, dd = double doublet, m = multiplet*.

**Table 3:** ^13^C NMR spectral data for **1, 2, 5–7, 9, 18, 19** in CD_3_OD at 25°C.

	1	2	5	6	7	9	18	19
***Aglycone***								
2	146.3	147.0^B^	149.0	149.3^B^		148.8^B^	148.8	148.8
3	137.1	137.4	137.1	137.1		137.1	136.9	137
4	177.1	177.6	177.5	177.5	177.5	177.6	177.3	177.5
5	147.0	153.15	146.8	147.0		147.0	146.8	147.5
6	130.3	133.3	130.9	130.9		130.7	129.7	132.1
6 (OCH_3_)				61.5				61.0
7	152.5	157.6	152.7	152.7		151.7	154.6	158.2
8	95.4	95.4	95.4	95.1		94.9	94.0	94.7
9	150.0	152.9	150.4	150.4		146.5	151.1	153.7
10	106.2	106.67	106.67	106.74		106.8	104.8	105.0
1′	123.9	123.9	123.7	124.1		123.9	124.4	124.1
2′	116.2	116.18^$^	130.9	116.4	133	116.2	116.0	116.0
3′	145.9	146.2	116.30^$^	146.0		146.1	146.2	146.1
4′	148.5	148.8	160.7	149.0		149.3^B^	148.7	148.7
5′	116.1	116.23^$^	116.30^$^	116.25^$^	122	116.2	116.2	116.2
6′	121.8	121.9	130.9	121.7	133	121.9	121.7	121.7
***Glycosyl***								
1′′	102.8	102.0	102.6	102.4		102.1		
2′′	74.4	74.6	74.7	74.8		74.5		
3′′	78	77.2	77.5	77.9		77.4		
4′′	71.1	71.3	71.3	71.3		72.0		
5′′	77.9	75.8	78.5	78.5		75.7		
6′′	62.7	64.2	62.50	62.55		64.6		
*6′′-O-acyl*		*6′′-O-galloyl*			*6′′-O-galloyl*	*6′′-O-caffeoyl*		
1′′′		121.3				127.2		
2′′′		110.1			110.1	115.6		
3′′′		146.4				146.4		
4′′′		139.8				149.2		
5′′′		146.4				116.4		
6′′′		110.1			110.1	122.2		
*A*						147.5		
*B*						114.4		
C = O		168.3				168.9		

*^$^These values are ranked from lowest to highest shift-values from HSQC. Exact shift-values were then determined from CAPT*.

### NMR Elucidation of Compounds

The downfield part of the 1D ^1^H NMR spectrum of **1** and **18** showed a 3H ABX system at 7.76 ppm (**1**: *dd* 2.0 Hz, 8.0 Hz; H-6′), 7.72 ppm (**18**: *dd* 3.6 Hz, 7.2 Hz; H-6′), 7.86 ppm (**1**: *d* 2.0 Hz; H-2′), 7.82 ppm (**18**: *d* 3.6 Hz; H-2′), 6.98 ppm (**1**: *d* 8.4 Hz; H-5′) and 6.97 ppm (**18**: *d* 7.8 Hz; H-5′), and an unresolved singlet at 7.03 ppm (**1**: H-8) and 6.58 ppm (**18**: H-8). In the HMBC spectrum of **1** the aromatic proton at 7.03 ppm showed correlations to the aromatic carbons at 106.2 ppm (C-10), 130.3 ppm (C-6), 150.0 ppm (C-9), 152.5 ppm (C-7), as well as to the carbonyl function at 177.1 ppm (C-4), revealing the aglycone to be 6-hydroxyquercetin (**Table 3**). The ^1^H and ^13^C NMR data of the glycone moiety was quite similar to those of quercetagetin (6-hydroxyquercetin; Abdala, 1999; Senatore et al., 1999; Aquino et al., 2002).The molecular mass at *m/z* 319.0435 of **1** and 319.0433 of **18** in their ESI high-resolution MS spectra were in accordance with 6-hydroxyquercetin (C_15_H_10_O_8_ + H^+^; calc: 319.0454).

The sugar region of the 1D ^1^H NMR spectrum of **1** showed the presence of one sugar unit by the single anomeric proton signal at 5.14 ppm (J = 7.4 Hz). In the COSY spectrum of this proton the observed crosspeak at 5.14/3.71 and the following crosspeaks were in accordance with seven sugar protons, which indicated that this sugar unit was a hexosyl. The corresponding crosspeaks in the HSQC spectrum permitted the assignment of H-2′′, H-3′′, H-4′′, H-5′′, H-6A′′ and H-6B′′, and the chemical shifts and coupling constants of **1** were in accordance with β-glucopyranosyl (Harborne et al., 1991; Parejo et al., 2005). A cross peak at δ 5.14/152.5 (H-1′′/C-7) in the HMBC spectrum confirmed the connection point of the sugar unit to be in the seven-position of the aglycone (Harborne et al., 1991). The molecular mass at *m/z* 481.0963 in the ESI high resolution mass spectrum of **1** was in accordance with 6-hydroxyquercetin 7-*O*-β-glucopyranoside (C_21_H_20_O_13_ + H^+^; calc: 481.0982).

**FIGURE 2:**
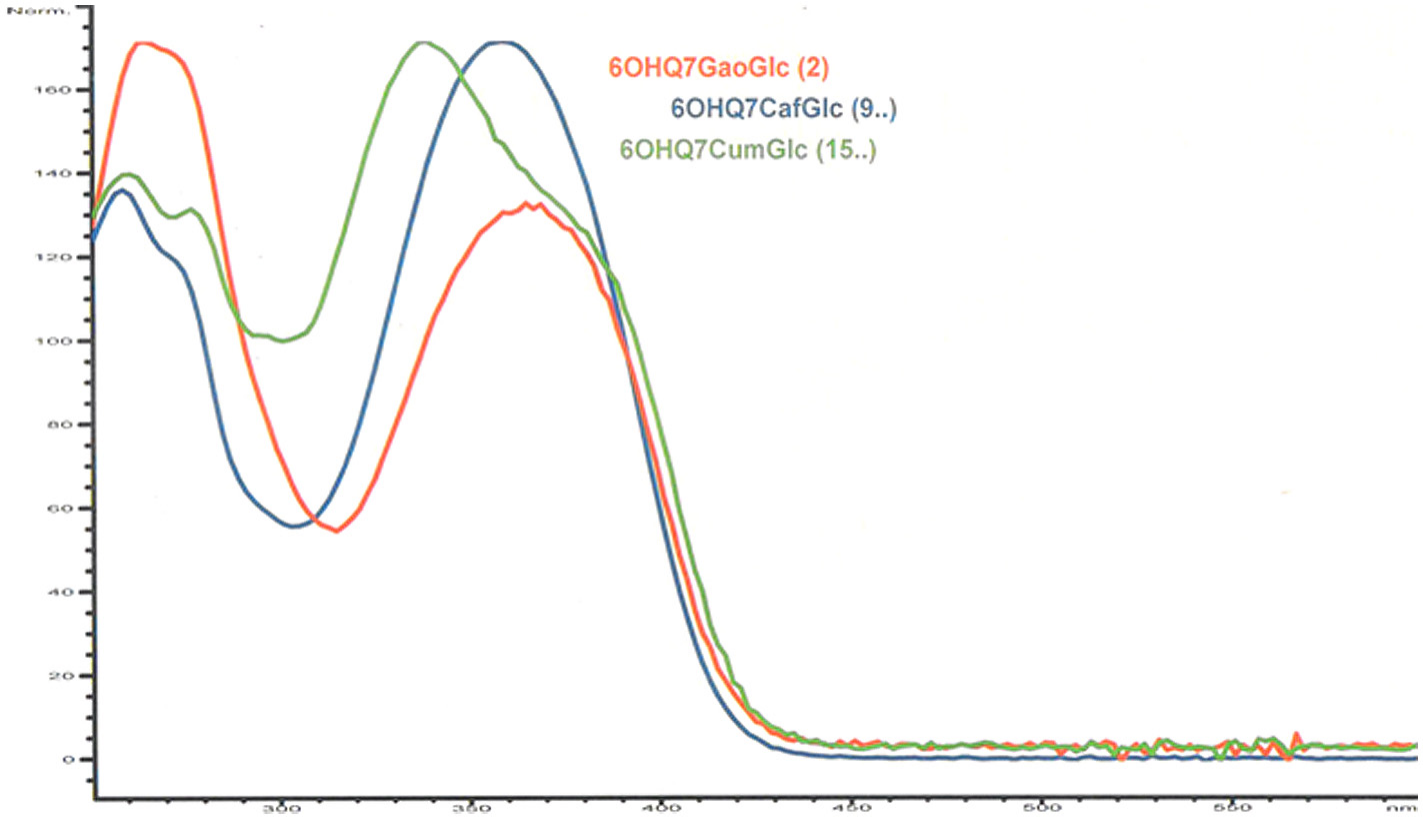
**UV spectra of **2, 9,** and **15** recorded on-line during HPLC analysis**.

The ^1^H NMR spectrum of flavonol **2** shared many similarities with the corresponding resonances of **1** (**Table 2**). However, the chemical shift values of H-6A′′ (4.78 ppm), H-6B′′ (4.55 ppm), H-5′′ (3.94 ppm) and C-6′′ (64.2 ppm) of the sugar, indicated the presence of acylation at the 6′′-hydroxyl. The two-proton singlet at 7.13 ppm of H-2′′′ and H-6′′′ in the 1D ^1^H NMR spectrum of **2** was in accordance with a galloyl unit (**Table 2**; Brakat et al., 1999; Chan et al., 2014). The crosspeaks at δ 4.78/168.3 (H-6A′′/C = O galloyl) and δ 4.55/168.3 (H-6B′′/C = O gallyol) confirmed the linkage between the 7-glucosyl and the galloyl moiety to be at the 6′′-hydroxyl (**Tables 2** and **3**; Brakat et al., 1999). **Figure 2** shows the UV spectra of the three compounds **2** with UV_max_ at 365 nm, **9** with UV_max_ at 359 nm and **15** with UV_max_ at 339 nm respectively. The molecular mass at m/z 633.1066 (C_28_H_24_O_17_ + H^+^; calc: 633.1092) in the high resolution MS spectrum of **2** was in accordance with 6-hydroxyquercetin 7-*O*-β-(6′′-galloylglucopyranoside).

The ^1^H and ^13^C resonances of the aglycone and monosaccharide of **9** were assigned by a combination of 1D ^1^H NMR, DQF-COSY, HSQC, and HMBC experiments (**Tables 2** and **3**) to be 6-hydroxyquercetin 7-*O*-β-glucopyranoside. The crosspeak at δ 5.18/151.7 (H-1′′/C-7) in the HMBC spectra confirmed the linkage between the aglycone and sugar unit to be at the 7-hydroxyl moity. The doublets at δ 6.83 (1.5 Hz; H-2′′′), δ 6.62 (8.1Hz; H-5′′′), δ 7.53 (15.8 Hz; H-*α*) and δ 6.27 (15.8 Hz; H-β), and the double doublet at δ 6.66 (*dd*, 7.8Hz, 1.8Hz; H-6′′′) in the 1D ^1^H NMR spectrum of **9** was in accordance with a caffeoyl unit. These data were consistent with the presence of a caffeoyl moiety (**Table 2**; Nair et al., 1993; Diaz and Herz, 2010). The crosspeaks at δ 4.74/168.9 (H-6A′′/C = O caffeoyl) and δ 4.42/168.9 (H-6B′′/C = O caffeoyl) in the HMBC spectrum confirmed that an acyl moiety was linked to be at the 6′′-hydroxyl (**Table 3**; Nair et al., 1993). The molecular mass at m/z 643.1272 (C_30_H_26_O_16_ + H^+^; calc: 643.1299) in the high resolution MS spectrum of **9** was in accordance with 6-hydroxyquercetin 7-*O*-β-(6′′-caffeoylglucopyranoside).

The UV spectra of **5** and **7** showed UV_max_ at 345 nm and 349 nm, respectively (**Table 1**). The 1D ^1^H NMR spectrum of **5** showed a singlet at 7.03 ppm (H-8) and a 4H ABXY system of two doublets at 8.21 ppm (*d*, 8.8 Hz; H-2′′ and H-6′′) and 6.99 ppm (*d* 8.9 Hz; H-3′′ and H-5′′) in accordance with the flavonol, 6-hydroxykaempferol (**Table 2**; Hattori et al., 1992). On the basis of signals in the 1D ^1^H NMR, ^1^H-^1^H COSY,^1^H-^1^H TOCSY,^1^H-^13^C HSQC ^1^H-^13^C HMBC spectra, the chemical shifts (^1^H and ^13^C) of **5** were in agreement with 6-hydroxykaempferol linked to one β-glucopyranose unit (Hattori et al., 1992). The crosspeak at 5.14/152.7 ppm (H-1′′/C-7) in HMBC spectra confirmed the linkage between the aglycone and the sugar unit to be at the 7-hydroxyl (**Table 3**). The molecular mass at *m/z* 465.1009 in the ESI-MS spectrum of **5** was in accordance with 6-hydroxykaempferol 7-*O*-β-glucopyranoside (C_21_H_20_O_12_ + H^+^; calc: 465.1033).

The chemical shift values in 1D ^1^H NMR spectrum of **7** were similar to those of **5**. It showed a singlet at 6.96 ppm (H-8) and a 4H ABXY system of two doublets at 7.9 ppm (*d*, 8.8 Hz; H-2′′ and H-6′′) and 6.92 ppm (*d* 8.9 Hz; H-3′′ and H-5′′) were in accordance with the flavonol, 6-hydroxykaempferol (**Table 2**). The ESI-MS spectra showed the fragment ion due to cleavage of the glycosidic bond, which suggested the presence of 6-hydroxykaempferol (*m/z* 303.0485). Some of the NMR signals were missing or very weak, but the singlet at 7.15 ppm of H-2′′′ and H-6′′′ in the 1D ^1^H NMR spectrum of **7** was in accordance with a galloyl unit (Brakat et al., 1999). The molecular mass at *m/z* 617.1137 in the high-resolution ESI-MS spectrum was in accordance with 6-hydroxykaempferol 7-*O*-β-(6′′-galloylglucopyranoside; C_28_H_24_O_16_ + H^+^; calc: 617.1143).

The 1D ^1^H NMR spectra of **6** and **19** revealed the presence of 6-methoxyquercetin aglycone. Each of their ^1^H NMR spectrum showed four aromatic proton resonances, a singlet at 6.91 ppm (**6**: H-8), 6.58 ppm (**19**: H-8) 7.52 ppm (**6**: *dd* 2.0 Hz, 8.5 Hz; H-6′), 7.72 ppm (**19**: *dd* 1.9 Hz, 8.0 Hz; H-6′), 7.82 ppm (**6**: *d* 2.0 Hz; H-2′), 7.82 ppm (**19**: *d* 1.9 Hz; H-2′), 6.90 ppm (**6**: *d* 8.6 Hz; H-5′) and 6.97 ppm (**19**: *d* 8.2 Hz; H-5′) along with a methoxy group at 3.98 ppm (**6**) and 3.97 ppm (**19**). The peaks assigned in ^1^H NMR corresponded to 6-methoxyquercetin (Andrade et al., 1999; Wei et al., 2011). The molecular mass at *m/z* 333.0597 of **6** and 333.0611 of **19** in the ESI high-resolution MS spectra were in accordance with 6-methoxyquercetin (C_16_H_12_O_8_ + H^+^; calc: 333.0610). ^1^H and ^13^C resonances of the monosaccharide of **6** were assigned by a combination of 1D ^1^H, DQF-COSY, TOCSY and experiments were in accordance with β-glucopyranosyl. The molecular mass at *m/z* 495.1137 in the ESI-MS spectrum of **6** was in accordance with 6-methoxy quercetin 7-*O*-β-glucopyranoside (C_22_H_22_O_13_ + H^+^; calc: 495.1139).

In literature, only a few reports dealing with compounds isolated from *Tagetes* species have addressed full structural elucidation of individual flavonoids including NMR assignments. Particularly no reference has been found with respect to NMR investigations of flavonoids in *T. minuta*. Abdala and Seeligmann (1995) and Tereschuk et al. (2004) have reported tentative occurrence of flavonols quercetagetin, patuletin, isorhamentin, and quercetin as aglycones with glycosides linkages without acylations in *T. minuta.* In the current study, we also identified 6-hydroxy- and 6-methoxyquercetin glycosides with acylation in *T. minuta*. 6-hydroxykaempferol was found in **5** (6-hydroxykaempferol 7-*O*-β-glucopyranoside) and **7** (6-hydroxykaempferol 7-*O*-β-(6′′ galloylglucopyranoside)) has been identified for the first time in *T. minuta.* The flavonols **2** (6-hydroxyquercetin 7-*O*-β-(6′′-galloylglucopyranoside)) and **9** (6-hydroxyquercetin 7-*O*-β-(6′′-caffeoylglucopyranoside)) have previously been reported only in extracts of *T. maxima* (Parejo et al., 2005), while this is the first report on complete identification of compound **7** in *T. minuta*.

### Antibacterial Activity

The antibacterial activity of crude, aqueous, ethyl acetate, and *n*-butanol extracts of different parts (flowers and seeds, roots, leaves, and stems) of *T. minuta* were tested against gram positive (*M. leteus, S. aureus, B. subtilis*) and gram negative (*P. pikettii, E. coli, S. setubal*) bacteria (**Table 4**). The isolated flavonols 1, 2, 5, 6, 7, 9, 18, and 19 were also subjected to antibacterial screening. All the samples showed significant antibacterial activity while these samples were inactive against *E. coli* and *S. setubal*. Activity comparison of three parts of plant indicated that flowers and seeds extract have statistically significant impact. Besides the extracts, only the isolated flavonols 1, 2 of butanol and 18 of ethyl acetate extract of flowers and seeds showed statistically significant (*p* < 0.05) activity against *M. luteus*. The crude extracts were statistically significant against gram positive (*B. subtilis* and *S. aureus*) and gram negative bacteria (*P. pikettii*). This study indicated that extracts and isolated flavonols of *T. minuta* have a broad spectrum of antibacterial activity.

**Table 4:** Zone of inhibition (mm) of success extracts and isolated compounds of *T. minuta* against different bacterial stains.

Microorganisms	Crude	Aqueous	Flower and seed extract		Leaves and stem extract		Root extract		Compounds			DMSO
			Ethyl acetate	Butanol	Ethyl acetate	Butanol	Ethyl acetate	Butanol	1	2	18	
*Micrococcus luteus*	0.0 ± 0.0^p^	0.0 ± 0.0^p^	6.3 ± 0.5^f^	1.0 ± 0.0^o^	0.0 ± 0.0^p^	1 ± 0.1^o^	0.0 ± 0.0^p^	0.0 ± 0.0^p^	19.0 ± 0.8^a^	14.2 ± 0.2^b^	10.0 ± 0.0^c^	0.0 ± 0.0^p^
*Staphylococcus aureus*	6.5 ± 0.4^ef^	0.0 ± 0.0^p^	4.3 ± 0.5^jk^	3.6 ± 0.0^l^	3.1 ± 0.2^lm^	0.0 ± 0.0^p^	3.0 ± 0.0^m^	2.3 ± 0.2^n^	0.0 ± 0.0^p^	0.0 ± 0.0^p^	0.0 ± 0.0^p^	0.0 ± 0.0^p^
*Pseudomonas pikettii*	5.0 ± 0.0^ij^	0.0 ± 0.0^p^	7.0 ± 0.8^de^	5.2 ± 0.2^hi^	6.3 ± 0.5^f^	0.0 ± 0.0^p^	5.0 ± 0.0^ij^	0.0 ± 0.0^p^	0.0 ± 0.0^p^	0.0 ± 0.0^p^	0.0 ± 0.0^p^	0.0 ± 0.0^p^
*Bacillus subtilis*	6.5 ± 0.4^ef^	5.7 ± 0.2^gh^	7.5 ± 0.5^d^	7.6 ± 0.2^d^	6.1 ± 0.2^fg^	5.5 ± 0.4^hi^	0.0 ± 0.0^p^	4.2 ± 0.2^k^	0.0 ± 0.0^p^	0.0 ± 0.0^p^	0.0 ± 0.0^p^	0.0 ± 0.0^p^

*Data represents ± mean standard deviation of triplicate. Means with common letters are not significantly different at *p* < 0.05 according to Duncan’s multiple-range test*.

The glycosilated and acylated flavonols of 6-hydroxyquercetin were strongly active in contrast to non-glycosilated flavonol. The results were in agreement with previous work of preliminary screening of the leaf extracts of *C. tergemina* displayed antibacterial activity against *M. luteus*, *B. cereus*, methicillin-resistant *S. aureus* containing acylated flavonol glycosides (Chew et al., 2011; Chan et al., 2014). The antimicrobial effect of crude flavonoids extracts has been reported in leaves of *T. minuta* (Tereschuk et al., 1997, 2004). The antibacterial activity of essential oil containing dihydrotagetone and ocimene has also previously been reported in *T. minuta* against Gram (+) and Gram (-) bacteria (Senatore et al., 2004). In this study antibacterial activity of plant extracts and purified acylated flavonol glycosides (1, 2, and 18) was reported for the first time in *T. minuta*.

## Conclusion

The extracts and purified compounds of plant were considered as suitable candidates for antibacterial drug discovery and support ethnopharmacological use, and its economical properties for commercialization of *T. minuta*. Based on these findings, we envision the discovery of new antibacterial agent from natural sources (plants) that will help to minimize the adverse effects of synthetic drugs.

## Conflict of Interest Statement

The authors declare that the research was conducted in the absence of any commercial or financial relationships that could be construed as a potential conflict of interest.
